# Identifying Differences in Frames of Reference That Are Hard to Reconcile During the Process of Normative Integration to Deliver Care for People with Multiple Problems: A Mixed-Method Delphi Study in the Netherlands

**DOI:** 10.5334/ijic.7583

**Published:** 2024-04-04

**Authors:** Lieke Reinhoudt-den Boer, Robbert Huijsman, Jeroen David Hendrikus van Wijngaarden

**Affiliations:** 1 Erasmus University, The Netherlands; 2 Erasmus University Rotterdam, The Netherlands; 3 Erasmus School of Health Policy and Management of the Erasmus University Rotterdam, The Netherlands

**Keywords:** integrated care, normative integration, people with multiple problems

## Abstract

**Background:**

Integrated care is enhanced by integration on system, organizational, professional, and clinical levels including functional and normative integration. Many studies have been done on functional integration on these different levels, less studies focus on how normative integration takes place. In this study, we focus on the question: what differences in frames of refence must be addressed to establish consensus on appropriate care for People with Multiple Problems?

**Methods:**

A mixed-method Delphi study was carried out in which professionals and managers regularly involved in care for people with multiple problems (PWMPs) worked towards consensus on appropriate care delivery through the assessment of 15 vignettes representing real trajectories of PWMPs.

**Results:**

No consensus on appropriate care delivery was reached on any of the 15 vignettes. Five differences in perspective explained the dissensus: 1) an individual versus a systemic perspective on the client; 2) a focus on self-expressed needs of clients or professionally assessed (normative) needs; 3) client-directed or caregiver-directed care; 4) client as victim of circumstances or responsible for circumstances; 5) a focus on barriers or opportunities.

**Conclusions:**

In general, panelists agreed that care for PWMPs should be integrated. However, the further integrated care was to be operationalized in practice the greater the dissensus between panelists emerged. To understand how these differences in perspectives may be overcome to provide care for PWMPs normative integration needs to be studied during actual processes of care delivery.

## Introduction

Integrated care is one of the cornerstones of policies aimed at improving care delivery for people with multiple problems (PWMPs) [1,2,3,4]. PWMPs are people who experience various combinations of problems such as: mental illness, intellectual disability, acquired brain injury, physical disability, behavioral difficulties, homelessness, social isolation, family dysfunction, and addiction [5]. It is widely acknowledged that people who have problems on psychological, mental, medical, and psycho-social levels need a continuum of care designed according to their multidimensional needs. This often implies that care is delivered by different actors, services and facilities involved on multiple levels of health and social care [6]. However, the landscape of health and social care is often fragmented and realizing integrated care continues to be complex and challenging [2, 7,8,9,10]. That is why PWMPs often do not get the support they need [8, 11].

Multiple studies suggest that the delivery of integrated care is enhanced by vertical and horizontal integration on system, organizational, professional, and operational levels [12, 13]. This not only requires the integration of systems and structures (functional integration), but also necessitates the integration of less tangible features related to social interaction (normative integration) [8, 12,13,14,15,16,17]. However, while many studies have been done on functional integration, much less have focused on normative integration [15,16,17,18,19].

Normative integration refers to aligning norms, values, and perspectives across organizations, professionals, and individuals on different levels in health and social care. It ensures coherence and consensus on appropriate care delivery across disciplines and settings guiding collaborative efforts [20,21,22]. Integrated care necessitates pooling of diverse expertise, sharing of uniquely held information and bridging the fragmented, specialized silos [16]. The clash of cultures and professional/functional-specific norms, values, and perspectives is one of the many reasons why integration efforts fail [15, 17]. Normative integration is viewed as one of the essential features in bridging these disparities and facilitating collaborative processes [10, 13, 15,16,17, 23]. Normative integration is expected to be stimulated by interdisciplinary group learning, defined as the development, modification, and reinforcement of frames of reference through processes of group interaction [15, 24].

Normative integration is an essential part of integrating care but is also an understudied phenomenon. There is a major call for more (practical) research on normative integration in literature on integrated care [8, 10, 15, 17]. The few studies that have been conducted on normative integration have provided insight into, for example, how normative integration can be measured or understood [15], how different actors have different perspectives on what values count in integrated care [8, 17, 18], or the potential effects of normative integration [16].

Aiming to contribute to literature on integrated care, this study examined a specific aspect of normative integration, namely “what differences in frames of refence must be addressed to establish consensus on appropriate care for PWMPs?”. It is regarded that achieving consensus on appropriate care for a particular (group of) clients is pivotal to normative integration. In contrast to the definition of Valentijn et al., which emphasizes the development and maintenance of a common frame of reference, including a shared mission, vision, values, and culture among actors on different levels [13, p.8, 16], we posit that normative integration involves upholding actors’ specific professional and functional norms, values and viewpoints while reaching consensus on how care should be tailored for a group of clients. The former is crucial for preserving the advantages stemming from diverse actors with different professional and functional norms, while the latter is crucial for fostering collaboration and aligning shared action which is essential to integrate care. Thus, our focus in this study is on identifying the disparities in frames of reference that must be overcome to reach consensus on appropriate care for PWMPs. To address our research question, we conducted a mixed-method Delphi study in which professionals and managers from various levels within health and social care worked towards consensus on appropriate care delivery through the assessment of 15 vignettes representing real care trajectories of PWMPs.

## Methods

The mixed-method Delphi study was structured to facilitate the consensus-building process among participants as it is specifically ‘designed as a group communication process which aims to achieve a convergence of opinions’ [25, p.1]. This technique helps participants to become more ‘problem-solving oriented, to offer their opinions more insightfully’, to minimize their focus on group or individual interests and thereby stimulates the convergence of opinions [25, p.2]. Given the focus of a Delphi study on structuring the consensus-building process, it offers an added advantage by effectively illuminating the differences in frames of reference that hinder this consensus-building process.

### Setting

This study was part of a larger study between September 2015 and November 2018 in Rotterdam, the Netherlands. This study started soon after a major welfare state reform was enacted in the Netherlands. The reform decentralised responsibilities for youth care, care for people with disabilities and psychiatric problems, long-term non-residential care for frail elderly, welfare policy for long-term unemployed and sheltered work for people with disabilities from the national government to municipalities. Likewise, responsibilities for contracting community nursing and activities of daily living assistance were placed under the responsibility of health insurers and responsibilities for residential care were transferred to regional care offices [26, 27, 28]. The overall study aimed to evaluate the suitability and level of integration of health and social care for PWPMs entering care trajectories via the municipality of Rotterdam. The idea behind decentralizing major aspects of social care and healthcare to municipalities was that municipalities are more capable than the national government of being responsive to local needs and can provide tailored, integrated care as they are (literally) closer to clients. These advantages would especially apply to and improve care for PWMPs whose needs span health and social issues [26, 28]. Rotterdam was an interesting setting to study care for PWMPs as it is second largest city in the Netherlands and is known for its large population of people with socioeconomic and (psycho)social problems [29].

### Participants

A purposeful sampling strategy was used to identify panelists with the following criteria for selection: representing one of the actor groups regularly involved in care for PWMP in Rotterdam, and who had more than 5 years of experience at professional, management, or system level in care for PWMPs. Participants working at different levels (professional, management and system level) were recruited as literature on normative integration suggests that normative integration spans system, organizational, professional, and clinical levels [1, 2, 30]. Normative integration is thus not only about integration among professionals or officers at similar levels, but also across levels. Informal caregivers were excluded as panelists. The larger study of which this study was a part had already shown that numerous PWMPs either lacked informal caregivers or their caregivers were unable to participate in the study.

The panelists were contacted through professional networks and invited to voluntarily participate in the project. A total of 12 panelists were approached by email and/or telephone and invited to participate. We then included 10 panelists that met our inclusion criteria and who indicated to be able to participate in the consensus rounds. Ten panelists are a recommended number of participants to ensure the development of a productive group dynamic and developing consensus among panelists [31, p.5]. After inclusion, all panelists received a telephone call and e-mail with study details and an additional message in the week leading up to each round. Information about the participating panelists can be found in Table 1.

**Table 1:** Characteristics of participating panelists.


TYPE OF PROFESSIONAL AND OFFICER	ORGANIZATION	PARTICIPATED IN WAVES

Team leader/Manager	Social care organization	1,2

Policy advisor long term care	Health insurer	1,2,3

Quality officer social care	Municipality of Rotterdam	1,2,3

Community-based nurse	Home care organization	1

Quality officer Dept Restructuring	Municipality of Rotterdam	1,2,3

Team leader/Manager	Social care organization	1,2,3

Team leader/Manager community-based primary care team	Municipality of Rotterdam	1,2,3

Policy advisor	Municipality of Rotterdam	1,2,3

Team leader/Manager	Mental health and addiction organization	1

Team leader/Manager	Home care organization	1


A total of 10 panelists participated in the first wave, 7 participated in the second wave, and 6 in the third wave. Panelists dropped out due to personal circumstances and unforeseen work obligations. In each wave, all panelists completed the questionnaires.

### Overview of data collection

**Figure 1:**
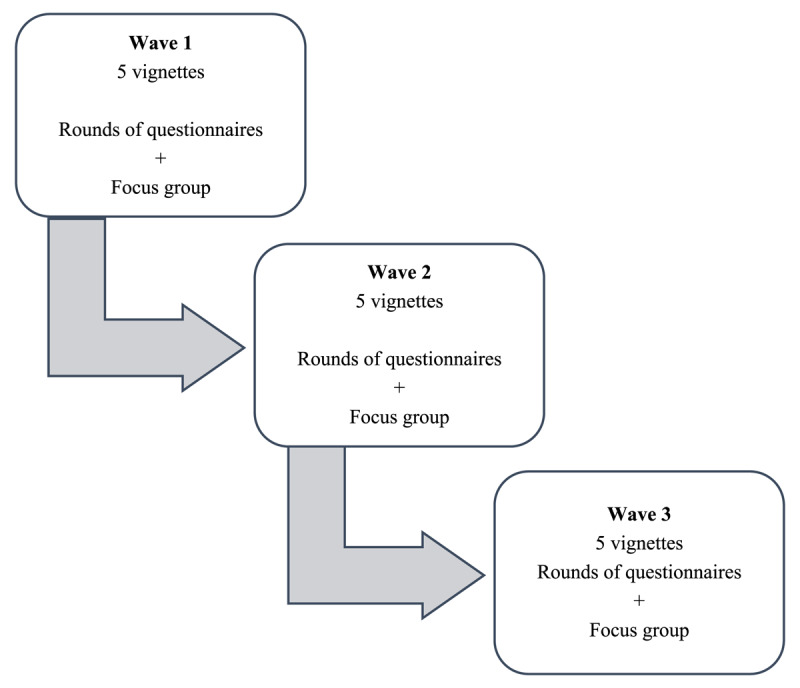
Overview of data collection process.

Data were collected in three waves (see Figure 1). In preparation for each wave the participating panelists received five vignettes representing real PWMPs’ care trajectories. Panelists then scored the five cases via self-administered questionnaires. Each wave was concluded with a focus group. Data were collected between June 2018 and November 2018. Our data collection strategy was carefully developed. The specific approach of our data collection using vignettes, self-administered questionnaires and focus groups structured the illumination of disparities in frames of reference and the consensus-building process. The use of vignettes ensured that all panelists had an identical information base, mitigating the possibility of discrepancies in frames of reference arising from variations in case-related information. The self-administered questionnaires served as an initial step in elucidating individual frames of reference crucial to consider during the consensus-building process regarding appropriate care for a client. It also enhanced the consensus-building process. The focus groups served as arena to address and discuss differences in frames of refence. The data were collected in 3 waves because it was too labor intensive for the panelists to review the 15 vignettes and discuss them all at one time. Although the three rounds were prompted by the labor intensity of reading and reviewing the vignettes, we also considered this as an additional step in the consensus-building and collective learning process toward normative integration about what constitutes appropriate care for PWMPs.

### Vignettes

We developed vignettes about PWMP care trajectories to present the whole client situation in the same way to very different professionals who all participated in one of more parts but not the whole of the care trajectory. The vignettes, covering around 15 pages each, provided thick descriptions about the PWMPs’ history (e.g., problems, prior care and support trajectories, and personal background), their living, work, family situations, and experienced problems. These descriptions also included their care trajectories over the course of 1,5 years: the care received by the PWMP and provided by (in)formal caretakers, content and frequency of interactions and communication among the PWMP and (among) involved (in)formal caretakers, their experiences with and perspective on the care received or provided, type, and involved (in)formal caregivers, and experiences and considerations regarding the care provided. The vignettes furthermore gave information on the outcomes achieved during the care trajectory. Consequently, panelists not only had the same information, but also had a lot more information than they normally have in real care trajectories in which they must collaborate to provide integrated care. The characteristics of PWMP and their caregivers involved can be found in Tables 2 and 3.

**Table 2:** PWMPs’ characteristics.


SEX	

**Male**	10

**Female**	5

**Total**	**15**

**AGE**	

**25–50 years**	6

**51–75 years**	6

**76–100**	3

**Total**	**15**

**LIVING CIRCUMSTANCES**	

**Alone**	8

**With partner/roommates/children**	5

**With partner/roommates & child(ren)**	2

**Total**	**15**

**TYPE OF PROBLEMS**	

**Finances (e.g., no income or debts)**	15

**Daytime activities (e.g., no daytime activities)**	10

**Housing (e.g., impending house eviction, homelessness, or contaminated house)**	9

**Domestic relationships (e.g., domestic violence or parenting problems)**	3

**Physical health**	9

**Mental health (e.g., mental problems or mental illness)**	14

**Addiction**	6

**Activities of daily living**	6

**Social network (e.g., absence of a social network or a destructive social network**	8

**Participation in society (e.g., no job or no volunteer work)**	10

**Encounters with law enforcement system (e.g., (pending) lawsuits for criminal activities)**	5


**Table 3:** Professional’ and informal caretakers’ characteristics


TYPE OF PROFESSIONAL CARETAKER	ORGANIZATION	NUMBER

**Informal caretakers**	N/A	6

**Community-based primary care team professional**	Municipality of Rotterdam	20

**Social worker**	Organization for addiction treatment Organization for people with acquired brain injury Religious social work organization Organization for sheltered living	7

**Psychiatric nurse**	Mental health organization Organization for addiction treatment	2

**Psychiatrist**	Mental health organization	1

**Trustee**	Trustee’s office	6

**Debt counselor**	Organization for forensic and specialized care Voluntary organization for debt counseling Debt counseling organization	3

**Spiritual caretaker**	Organization for spiritual care	1

**Pro bono legal counselors**	Municipality of Rotterdam	1

**General Practitioner**	General practice	2

**General-practice-based nurse specialist specialized in mental health**	General practice	1

**Social support act professionals responsible for assigning care for which an indication from the municipality was necessary (in Dutch Wmo-consulenten)**	Municipality of Rotterdam	2


Data for the vignettes consisted of multiple interviews with PWMPs, multiple interviews with involved (in)formal caretakers and information recorded about PWMPs in the municipal record system. PWMPs were interviewed three times at an interval of six months (T0, T1, T2). The first interview was held shortly after the start of the care trajectory (T0). During this period (in)formal caretakers were interviewed three times (T0, T1, T2). And formal caretakers of 15 clients were interviewed two times (T1, T2). Data in the municipal record systems about participating PWMPs were also part of this study’s data. PMWPs including their informal caretakers were followed 1 to 1,5 years. Based on the available data, the first author developed a draft vignette. After the first and third authors reached intercoder agreement that the vignettes represented the available data adequately, the vignettes were shared with the research group consisting of representatives of the university and the municipality of Rotterdam. Based on their feedback, the vignettes were refined and finalized.

### Self-administered questionnaires

All panelist evaluated the vignettes first individually by answering a pre-structured questionnaire. The questionnaire was co-created with representatives from the municipality focusing on the main goals of the decentralization, as formulated by the central government and adapted by the municipality of Rotterdam. Based on the input and discussions with these representatives some definitions in the questionnaire were expanded and questions were added. Specifically questions number 4, 18 and 19 were added as these relate to important policy goals of the municipality (see appendix I). In each questionnaire the participating panelists evaluated the level to which care was attuned to the PWMP’s multidimensional needs, the level to which care was designed and delivered coordinated ensuring a continuum of care, and the outcomes of the care trajectory. The draft questionnaires were discussed and pretested in several Delphi rounds with representatives of the municipality and revised until consensus was reached. The questionnaire consisted of 19 items using a five-point Likert scale ranging from totally not agree, not agree, neutral, agree, totally agree. The participating panelists could also motivate their answers using open text boxes. The questionnaire can be found in Appendix I. The link to the questionnaires was sent to the panelists by e-mail two weeks prior to each Delphi round. First, all panelists received the average scores per item and other panelists’ motivations for their scores on items. Second, they had the opportunity to adjust their own scores. We aimed to repeat this until at least 70% agreement was reached or no more changes to scores were made. Non-consensus was assumed if participant made no major changes nor suggestions for changes after a minimum of two rounds of questionnaires and a focus group.

### Focus groups

After each wave in which five vignettes were scored via the questionnaires, a focus group was held. During the focus groups the outcomes of the questionnaires were discussed. The aim was to identify underlying frames of reference that hinder consensus building (normative integration). The three focus groups were led by the first and third authors. During the focus groups, the authors enhanced mutual understanding via exchange of values, perspectives and interpretations, shared learning, and developing a shared perspective on appropriate care delivery under guided circumstances [20]. After each focus group the participating panelists were given the opportunity to adjust their scores when the plenary session had led to a shift in their opinion. All focus groups were recorded and transcribed verbatim.

### Data analysis

Responses to each round of questionnaires were tallied and frequencies were calculated. Descriptive statistics were used to describe structured responses. The data from the focus groups were analyzed by the first and third authors. In this step special attention was given to the discussion in the focus groups to identify the differences in frames of reference. For example, during the focus group, a debate emerged regarding the definition of the client: whether it should be solely the individual PWMP or encompass the PWMP along with their informal support network. This particular focus influenced how panelists made scoring decisions for specific cases and was identified as a (potential) frame of reference.

Data were analyzed using the technique for thematic analyses of Luborsky [32]. This process entailed that the first and third author read the transcripts to get acquainted with them. Then, a second reading was conducted at which notes were made and preliminary themes were identified (open coding). The authors discussed their notes and preliminary themes and came to mutual agreement on an initial set of themes (axial coding). Next, each author independently coded the three transcripts using the initial set of themes as a guideline, although this could be modified and added while analysis proceeded. The two researchers shared and discussed their independent interpretations and codes to come to consensuses on the interpretations. Conflicting or incompatible interpretations were solved. Data were analyzed using Atlas.ti version 9.

### Ethics

The Ethics Review Board confirmed that our study was outside the scope of the Netherlands’ Medical Research Involving Human Subjects Act and that the rights and privacy of study participants were sufficiently considered (MEC-2017-348).

## Results

In this result section, we will first outline the outcomes of the questionnaire rounds followed by the results of the focus groups.

### Questionnaire rounds

Tables 4 and 5 give an overview of the consensus scores. It shows that during the questionnaire rounds consensus was not reached. As the consensus scores in each wave hardly changed when panelists were shown one other’s scores including justifications, it was decided to limit the number of questionnaire-rounds to two.

#### Consensus scores on items

**Table 4:** Consensus and non-consensus scores.


	FIRST WAVE		SECOND WAVE		THIRD WAVE	
		
	ROUND 1	ROUND 2	ROUND 1	ROUND 2	ROUND 1	ROUND 2

**Overall consensus score**	55% On average consensus on 5 items per case	56% On average consensus on 5 items per case	60% On average consensus on 7 items per case	60% On average consensus on 7 items per case	61% On average consensus on 4 items per case	61% On average consensus on 4 items per case

**Average number of items on which consensus was reached**	5 items	5 items	7 items	7 items	4 items	4 items

**Average number of items on which no consensus was reached**	14 items	14 items	12 items	12 items	15 items	15 items


#### Consensus scores on cases

**Table 5:** Consensus scores of cases.


CASE NUMBER	AVERAGE CONSENSUS SCORE ROUND 1	AVERAGE CONSENSUS SCORE ROUND 2

**1**.	55%	55%

**2**.	64%	66%

**3**.	51%	53%

**4**.	58%	58%

**5**.	50%	47%

**6**.	68%	68%

**7**.	49%	49%

**8**.	58%	58%

**9**.	69%	69%

**10**.	54%	54%

**11**.	59%	59%

**12**.	60%	60%

**13**.	59%	56%

**14**.	67%	67%

**15**.	61%	61%


We additionally tried to identify other scoring patterns. We checked the data for consensus scores on items, for example if on certain items consensus was reached over multiple cases. We also further analyzed consensus scores on clusters of items. For example, we checked if consensus was reached on the level to which care was attuned to the PWMP’s multidimensional needs, the level to which care was designed and delivered coordinated ensuring a continuum of care, and the outcomes of the care trajectory. However, no patterns in consensus scores were found, although we did find individual scoring patterns in which some panelists were generally more positive or negative than others. Appendix II gives an overview of how the cases were scored including the average consensus scores per item.

### Focus groups

After each wave in which the panelists scored five vignettes in two rounds of questionnaires, a focus group was held. Although these meetings resulted in some general shared views on all cases such as that care for these people should be integrated, consensus on individual cases was not reached, even after three waves.

We identified five basic differences in perspective between our panelists that seem to explain this lack of consensus, namely 1) an individual versus a systemic perspective on the client; 2) a focus on self-expressed needs of clients or professionally assessed (normative) needs; 3) client-directed or caregiver-directed care; 4) client as victim of circumstances or responsible for circumstances; 5) a focus on barriers or on opportunities.

#### Individual or systemic perspective on the PWMP

One of the most basic perspectives in which our panelists varied was whether the focus should be on the individual PWPM or on the system (including informal network) of which the PWPM is part of. The discussion about C12’s case illustrates this:

*“C12 is a homeless, addicted man without any income, is not registered anywhere and has no ID. He lives with his mother who has severe mental illness herself and his brother who is physically disabled. Another brother of C12 lives around the corner. This brother also has severe problems and tells C12’s mother often “not to let that bum live with her.” All members of the family have their own individual professional caretakers.”*

Some panelist focused only on the needs of C12 and if the care and support that was given was in line with these needs. Other panelists believed there should be an integrated approach for this family system. However, for some that only referred to the mother and brother, as part of the household, others also included the brother living around the corner. Panelists who took an individual perspective were often more positive about the care that had been given to the clients in our cases.

#### Focus on self-expressed needs or professionally assessed (normative) needs

Another difference in perspective was their conception of the PWMP’s needs. Most PWMPs entered the support trajectory with one straightforward request for help, e.g., to solve their homelessness, solve their debts, avert a pending house eviction and/or help with getting an income. While the caregiver always suspected or identified multiple problems. Panelists varied on whether the PWMP’s self-expressed needs or these professionally assessed needs should primarily guide the care process. The discussion about C15’s illustrates these different perspectives:

*“C15 is a man with severe alcohol and cannabis addiction, and he is in danger of being evicted from his house because he has not paid his rent for a long time. Initially, C15 wants help with averting the house eviction, and secondarily he wants help with his addiction (not with cannabis addiction). With help of several professional caregivers, he can stabilize his financial situation, avert his house eviction and he is able to overcome his alcohol addiction.”*

Some panelists were very positive about how well C15’s needs were addressed. They mentioned that *“C15 was satisfied with the help he got”* and *“he got (successful) help to improve his situation in terms of his financial situation and alcohol addiction [things he wanted to be helped with]”*. However, other panelists emphasized that C15 was still addicted to cannabis, had not improved in social participation (C15 still had no structural daytime activities, volunteer nor paid work), nor expanded his informal network (C15 did not express this as a need), and underlying problems were not diagnosed (e.g., some panelists suspected mild intellectual disability). Panelists who focused more on the professionally assessed needs, were often far less positive about cases, than panelists who put the PWMP’s self-expressed needs at the center.

#### Client-directed or caregiver-directed care

Another variation in the panelists’ perspectives on cases was whether PWMPs (including their informal network) should always be stimulated to self-direct their care-process. And consequently, differences in opinions occurred if responsibility and initiative for the care trajectory should be with the client (client-directed care) or with the caregiver (caregiver-directed care).

*“C14 (in his twenties) works as a postman, moved out from his parents’ home as a teenager after troubles with his parents, is homeless (sleeps at friends’ sofas or in a bus from work), has debts (C14 has no idea how much, as he hasn’t had an official postal address for a couple of years), has lived on his own but lost his homes several times as he appeared unable to meet the obligations associated with a house. He reaches out for help to get a home and a sufficient income. When he gets a professional caretaker, C14 hopes he will take over charge to solve his problems, and e.g., arrange a municipal postal address, arrange social benefits to supplement his income as a postman, arrange a home (or more permanent place to stay overnight) and take initiative to solve his debts. But his professional caretaker does not take over charge. He wants to help C14 but expects C14 to take initiative and do himself as much as possible.”*

When C14’s case was discussed, some panelists believed that the professional caretakers’ approach was adequate; C14 expects his professional caretaker to do the hard work, but it is appropriate to let C14 do as much as he can himself with help of his friends. One panelists notes: *“The assistance actually consisted of support and not of ‘taking over’ actions. C14 was therefore sufficiently stimulated to take/keep/gain control, but he himself had different expectations.”* Another panelist did not agree and suggested that professionals too readily believe that self-directed care is always better. However, they should pay more attention to the actual capabilities of clients at that moment. In his view *“no account is taken of (in)capability of the client”* and *“client’s abilities are overestimated”*, therefore the care and support delivered were not adequate.

#### Client as victim of circumstances or responsible for circumstances

Related but separate to the former, is a difference in perspective on seeing the client as a victim of circumstances or responsible for these circumstances. Some PWMPs in this study were involved in illegal activities, e.g., criminal activities, fraud with social services, or displayed difficult or aggressive behavior. Some panelists viewed illegal activities or aggressive behavior as part of the PWMP’s problem for which care and support are needed. Others however focused more on the PWMP’s accountability and personal responsibility. Discussions about C6’s vignette illustrates this. One panelist noted: *“Insufficient attention has been paid to personal responsibility, too much help like he’s a victim. While there are several indications of fraud (address in another city, concealed income…). The approach is too soft.”* Another panelist noted: *“C6’s situation has not been properly mapped out (language skills, mental abilities (possible mild mental disability) or brain damage), which gives me the idea that too much was asked of C6 which reinforced his aversion to care.”*

#### Focus on barriers or opportunities

A more general difference be/tween our panelists was their inclination to focus either on barriers or on opportunities, especially when asked about continuity of care and outcomes.

One group of panelists focused more on the barriers in the cases outlined in the vignettes. When evaluating the outcomes of the care trajectories they evaluated the outcomes given the specific circumstances. They for example highlighted that *“not all problems were solved, but what the caregivers did was the highest attainable”; “caregivers could coordinate the care more, but the man was difficult to help [displayed complex behavior in which he attracted and repelled caregivers]. More coordination might not have led to improved outcomes.”; “The man got basic care [after he got evicted, he got a new house and his financial situation was stabilized], his underlying problems were not addressed, but not more could be expected more in this vignette.”* Another group of panelists focused on the potential maximum outcomes if things were handled differently in the cases. They for example highlighted that *“the care process did not lead to more insight into underlying problems.”; “The man is 61 years he has still many years ahead, focus should have been on behavioral change, increasing his informal social support system as a safety net, that did not succeed.”; “more insight into his capabilities could have been gained via a psychological examination”; “the care trajectory should have been planned more consciously via e.g., the principle of stepped care.”* Panelists who focused on the barriers were often more positive about cases than panelists who focused on the opportunities.

## Discussion

Our aim was to study what differences in frames of reference must be addressed to establish consensus on appropriate care for PMWPs. We structured the process via a Delphi approach with multiple rounds and waves, structured criteria (based on research and current policy), and detailed case descriptions (vignettes). Our findings suggest that there are at least five important differences in frames of reference that form barriers for normative integration related to the delivery of health and social care for PWMPs, namely 1) an individual versus a systemic perspective on the client; 2) a focus on self-expressed needs or professionally assessed needs; 3) care as client-directed or caregiver-directed; 4) client as victim of circumstances of responsible for circumstances; 5) a focus on barriers or opportunities.

At a more abstract level, all panelists shared certain frames of reference such as the belief that integrated care is a valuable pursuit and that care for PWMPs is ideally delivered in an integrated fashion. However, participants disagreed when it came to the specifics, then even basic conceptions were challenged such as who is the client (perspective 1), what needs should guide the care trajectory (perspective 2), who must take the lead (perspective 3), what are the clients’ own responsibilities (perspective 4)? These differences seem to relate to individual preferences, professional education and experience, position, and institutional structures, rules, and policies [15, 17]. Although this is to our knowledge the first paper to study normative integration for these particular clients and these professionals and officers, other studies seem to suggest similar differences. For example, studies on collaboration between social workers and health care workers suggest that social workers put much more focus on autonomous decision making of clients and use a more systemic philosophy (perspective 1 and 2) [33, 34]. Differences in frames of reference may also relate to the position a person has in a system and the dominant frame of reference within that system [16]. In the last decade, in line with many European countries, the Dutch system made a shift from a welfare state to what is called a ‘participation society’ [35, 36]. Influenced by the necessity to balance the need to expand health and social services because of a growing (elderly) population and the imperative to curb public spending, a shift was made from inclusive solidarity towards exclusive selectivity, from collective responsibility towards individual responsibility [37, 38, 39]. Especially, in the city of Rotterdam with a right-wing counsel after decades of left-wing counsels, this shift was more pronounced than in most other Dutch cities. It is likely that officers who are involved in policy making or management, will more strongly relate to this frame of reference and thereby focus more on the individual responsibility of clients (perspective 3 and 4). While professionals directly working with these types of clients will probably more experience the barriers for these clients to be responsible and take responsibility. Finally, differences in frames of reference may not only relate to a particular profession or position but can also reflect individual differences based on character [40]. For example, some people are more inclined to focus on opportunities, while others focus more on the barriers (perspective 5).

The study’s outcome, highlighting the lack of consensus, prompts a need for reflection on the concept of normative integration. First, there is a fundamental question that needs addressing: to what extent does the absence of normative consensus pose a problem? The essence of integrated care lies in organizing services around the complex needs of clients with a diverse group of professionals or organizations, each contributing unique expertise that, when combined, can significantly benefit the client (insert reference). Consequently, integrated care should involve recognizing and valuing the differences among professionals from different disciplines and backgrounds. Current literature on normative integration seems to be too much focused on integration and too little on celebrating differences. Our study has also shed light on another facet of normative integration that warrants contemplation: the extent to which normative integration remains static outside the realm of action and how much it evolves during action. Providing care for people with complex needs, such as PWMPs, involves an ongoing process of sense-making within unfamiliar, uncertain, and multifaceted contexts, dealing with what is termed as normative complexity (Peeters and Oldenhof, 2023 – normative complexity). Based on our study, we speculate that normative integration predominantly takes shape during action, and is consistently reshaped, rather than existing as a static construct. Reflecting on our findings, we suspect that one of the reasons why consensus was not achieved was the rational and clinical nature of the Delphi process we organized, with basically nothing at stake for the participants and also no real possibility to test conceptions about the client and the professionals who actually delivered the care. Although the participants became familiar with each other’s frame of reference, and we were able to identify relevant differences, they had no way of testing if a certain frame better fits actual practice or led to a better result in actual cases. Also, as they had ‘no skin in the game’, there was no need to compromise or come to a consensus. It seems that normative integration is not only about shared reflection as some authors suggest, but also about shared action. Based on our experiences, we come to the understanding that building a common frame of reference is an iterative collective learning process in practice, as suggested in the (somewhat scarce) available literature on successful processes of normative integration and shared mental models [14, 20, 41, 42]. This process might be better supported by giving professionals the shared responsibility to work together and make decisions on actual cases, then by reflecting on example cases. Studying normative integration in action might also give us a better understanding of how differences in perspectives are negotiated in ‘real life’ settings. The different perspectives identified in this study might furthermore be used to make professionals, policy-workers and managers aware of their differences, which could create understanding and structure their discussions.

While our study has concentrated on normative integration among professionals, it is crucial to acknowledge that normative integration does not solely pertain to formal actors within the system, organizations, professional realms, and clinical settings (Evans, 2014; Kaehne, 2020; Oksavik et al., 2021; Kerrissey et al., 2022). There remains a considerable need for additional studies on normative integration involving clients and informal caregivers. Gaining deeper insights into their frames of reference is particularly essential to comprehend their perspectives and find effective methods for addressing differences.

Another important aspect on which we need to reflect is the use of a Delphi study. Although we used the Delphi study in a way not often done before: to study differences during the consensus-building process, we still think it was the best method for our research objective. At the same time, it is important to acknowledge potential drawbacks associated with employing a Delphi study. First is the dependency on expert opinion and the engagement of panelists during multiple rounds. We were confronted with a nurse who only participated in focus group one, leading to an emphasis on people with social care background and people working at officer level. This could have restricted the breath and insights and perspective considered in this study. Moreover, our designed Delphi study demanded significant time commitment from participants. Engaging in this study necessitated panelists to dedicate substantial hours to read and assess the 15 vignettes, partake in focus groups, and adapt their evaluations based on interactions with other panelists. Consequently, scaling up this study to include multiple groups of similar professionals and officers became unfeasible due to the resource-intensive nature of participation.

In addition, this study was highly embedded in a specific context (Rotterdam, the Netherlands) and involves a specific group of professionals, officers, and client group. The specific context and the specific group of professionals and officers could have affected its outcomes and the generalizability of our findings to other settings. Further research with other client groups, professionals and officers may provide a more comprehensive overview of differences in frames of reference that must be addressed.

## Conclusion

Our study outlines five dominant differences in perspectives that hinders normative integration between professionals to integrate care to people with multiple problems. At a high level of abstraction panelists had a common frame of references, however, the further integrated care was operationalized the greater their differences and the non-consensus became. More insight into normative integration in actual practice is required to understand how professionals and officers deal with these differences in perspectives.

## Additional File

The additional file for this article can be found as follows:

10.5334/ijic.7583.s1 Appendices. Appendix I and II.
