# Supplementary material for: Identifying Differences in Frames of Reference That Are Hard to Reconcile During the Process of Normative Integration to Deliver Care for People with Multiple Problems: A Mixed-Method Delphi Study in the Netherlands
Source: Int J Integr Care. 2024 Apr 4;24(2):2. doi: 10.5334/ijic.7583 (PMC11012147; doi:10.5334/ijic.7583)
Supplement: Appendices. — Appendix I and II. [file ijic-24-2-7583-s1.pdf]

Appendix I

Question	Totally not agree	Not agree	Neutral	Agree	Totally agree
1. The care provided is sufficiently attuned to the (multidimensional) needs of the client					
2. The right care is provided.					
3. The right choices are made concerning the usage of formal and informal care so that care is adequate but not too expensive.					
4. While providing care the right priorities are set.					
5. The right care is offered at the right time.					
6. The care provided is sufficiently attuned to the client's needs.					
7. The care provided supports the client’s informal network sufficiently so that overburdening is prevented.					
8. The care provided stimulates and supports the clients with multiple complex needs sufficiently so that s/he can take control over his/her live (considering the clients’ abilities, disabilities, and informal network)					
9. The care provided to the client is sufficiently coordinated across different providers and settings around client’s needs (delivered in an integrated manner).					
10. Formal caregivers have sufficiently discussed and coordinated the care needed for this client					
11. Information concerning this client has been shared sufficiently and timely among formal caretakers.					
12. The client has been referred at the right times to the right (most adequate) (in)formal caregivers.					
13. The right (in)formal caregivers and services were available at the right time for this client.					
14. The client’s informal network is sufficiently involved by the formal caregivers.					
15. The available care from different sectors is sufficiently coordinated around the client’s needs.					
16. The care provided has helped to solve or decrease one or more of the client’s problems.					
17. The care provided to this client has contributed sufficiently to optimizing the client’s self-reliance (considering the client’s capacities, disabilities, and social network).					
18. The care provided has sufficiently contributed to improving the client’s self-reliance.					
19. The care provided has contributed sufficiently to optimizing the client’s social participation.					

Appendix II

Average consensus per item

Question	Round 1	Round 2
1. The care provided is sufficiently attuned to the (multidimensional) needs of the client	60%	61%
2. The right care is provided.	66%	67%
3. The right choices are made concerning the usage of formal and informal care so that care is adequate but not too expensive.	58%	60%
4. While providing care the right priorities are set.	61%	64%
5. The right care is offered at the right time.	62%	62%
6. The care provided is sufficiently attuned to the client's needs.	60%	59%
7. The care provided supports the client’s informal network sufficiently so that overburdening is prevented.	51%	51%
8. The care provided stimulates and supports the clients with multiple needs sufficiently so that s/he can take control over his/her live (considering the clients’ abilities, disabilities, and informal network)	58%	58%
9. The care provided to the client is sufficiently coordinated across different providers and settings around client’s needs (delivered in an integrated manner).	62%	61%
10. Formal caregivers have sufficiently discussed and coordinated the care needed for this client	59%	60%
11. Information concerning this client has been shared sufficiently and timely among formal caretakers.	55%	54%
12. The client has been referred at the right times to the right (most adequate) (in)formal caregivers.	59%	60%
13. The right (in)formal caregivers and services were available at the right time for this client.	59%	58%
14. The client’s informal network is sufficiently involved by the formal caregivers.	55%	55%
15. The available care from different sectors is sufficiently coordinated around the client’s needs.	48%	49%
16. The care provided has helped to solve or decrease one or more of the client’s problems.	69%	69%
17. The care provided to this client has contributed sufficiently to optimizing the client’s self-reliance (considering the client’s capacities, disabilities, and social network).	61%	60%
18. The care provided has sufficiently contributed to improving the client’s self-reliance.	63%	63%
19. The care provided has contributed sufficiently to optimizing the client’s social participation.	52%	53%
